# Naringenin restores colistin activation against colistin-resistant gram-negative bacteria *in vitro* and *in vivo*

**DOI:** 10.3389/fmicb.2022.916587

**Published:** 2022-08-03

**Authors:** Mengxin Xu, Zhuocheng Yao, Yining Zhao, Shiyi Shi, Yao Sun, Luozhu Feng, Cui Zhou, Xiaodong Zhang, Jianming Cao, Tieli Zhou

**Affiliations:** ^1^Key Laboratory of Clinical Laboratory Diagnosis and Translational Research of Zhejiang Province, Department of Clinical Laboratory, The First Affiliated Hospital of Wenzhou Medical University, Wenzhou, China; ^2^Department of Medical Laboratory Science, School of Laboratory Medicine and Life Science, Wenzhou Medical University, Wenzhou, China

**Keywords:** colistin resistance, synergistic effect, naringenin, biofilm, gram-negative bacteria

## Abstract

Colistin is used as the “last line of defense” against multidrug-resistant (MDR) Gram-negative bacteria (GNB). However, improper use of colistin may further lead to an increasing number of colistin-resistant (Col-R) strains worldwide, which greatly limits antibiotic treatment options. In this study, we investigated the antibacterial and antibiofilm activities of naringenin (NG) combined with colistin against Col-R GNB *in vitro* and *in vivo*. The checkerboard method and time-kill test showed that NG combined with colistin has better antibacterial activity (FICI < 0.5) compared with NG and colistin alone. Biofilm formation inhibition tests demonstrated that combining the two drugs could inhibit biofilm formation; scanning electron microscopy (SEM) confirmed that the combination of the two significantly reduces the number of cells in the biofilm compared with the drug alone. The *in vivo* experiment showed that the combination of NG and colistin can improve the survival rate of the *Galleria mellonella* (*G. mellonella*) and reduce the microbial load in the mouse thigh infection model. Mechanistically, the combination of NG and colistin synergistically enhances the antibacterial activity and changes the permeability of the bacterial outer membrane. More importantly, cytotoxicity tests showed no cell cytotoxicity of NG in combination with colistin. In conclusion, our data revealed that NG combined with colistin exhibited good synergistic effects *in vivo* and *in vitro*, thus providing a new therapeutic option for clinical Col-R GNB infections.

## Introduction

Antibiotic resistance and the spread of multidrug-resistant (MDR) pathogens pose a serious threat to global health (El-Sayed Ahmed et al., 2020; Antimicrobial Resistance Collaborators, 2022). Colistin (polymyxin E) has re-emerged as a last-resort treatment option for bacterial infections, especially Gram-negative pathogens (Falagas and Kasiakou, 2005; Kaye et al., 2016). Yet, the improper use of colistin may further lead to an increasing number of colistin-resistant (Col-R) strains worldwide, which greatly limits antibiotic treatment options (Antoniadou et al., 2007; Liu et al., 2016). Over the years, several Col-R Gram-negative bacteria (GNB) have emerged worldwide and LPS, efflux pump, and PmrAB PhoPQ two-component system mutation have been considered the main factors regulating colistin resistance (Cai et al., 2012; Olaitan et al., 2014; Zhang et al., 2021a). A combination of natural compounds and antibiotics has been considered the main treatment option for Col-R GNB.

Colistin combined with fosfomycin, tigecycline, carbapenems, and rifampicin has shown a significant synergistic effect on treating GNB (Nordqvist et al., 2016; Abdelsalam et al., 2018; Wang et al., 2018a; Sato et al., 2021). Compared with synthetic antibiotics, phytochemicals have low toxicity and are not expensive. To date, resveratrol, eugenol, and astragalus, in combination with colistin, have been approved by the FDA for treating Col-R GNB (Cannatelli et al., 2018; Wang et al., 2018c; Zhou et al., 2018).

Biofilm formation is one of the main mechanisms through which bacteria develop antibiotic resistance. Biofilms are embedded in self-produced polymers such as polysaccharides, proteins, nucleic acids, and lipids, which maintain the stability of biofilms, mediate their adhesion to material surfaces, and help microorganisms colonize human organs or medical devices (Flemming and Wingender, 2010). In recent years, with the widespread use of antimicrobial drugs, nosocomial infections caused by biofilm-forming GNB, such as pneumonia, urinary tract infections, endocarditis, wound infections, and bacteremia, have substantially increased (Dijkshoorn et al., 2007; Prowle et al., 2011). Therefore, it is imperative to find effective drugs that can inhibit biofilms.

Recent studies have also shown that naringenin (NG), a natural flavonoid compound found in fruits and vegetables, may be used as a potential immunomodulator to treat sepsis, fulminant hepatitis, fibrosis, and cancer (Zeng et al., 2018). A recent study showed the synergistic effect of NG in combination with ceftazidime against ceftazidime-resistant *Enterobacter cloacae* (Eumkeb and Chukrathok, 2013). However, it remains unclear whether NG can be used in combination with colistin to treat Col-R GNB. In the present study, we evaluated the antibacterial and anti-biofilm effects of NG combined with colistin against GNB with different colistin resistance mechanisms and different strains, including *Klebsiella pneumoniae*, *Escherichia coli*, and *Acinetobacter baumannii in vitro*. In addition, we performed an *in vivo* experiment to further explore the synergistic effect of NG and colistin combination in the *G. mellonella* infection model and a mouse infection model.

## Materials and methods

### Bacterial isolates and growth conditions

In this study, 21 Gram-negative clinical Col-R isolates, including *K. pneumoniae* (*n* = 7), *E. coli* (*n* = 7), and *A. baumannii* (*n* = 7), were isolated from the First Affiliated Hospital of Wenzhou Medical University during 2015–2017. Strains were collected from different patients. Mechanisms of colistin resistance were identified by the experimental team (Chen et al., 2020; Zhang et al., 2021c). Identification of isolates was performed using the matrix-assisted laser desorption/ionization time-of-flight mass spectrometry (MALDI-TOF-MS, BioMerieux, France). Upon collection, isolates were snap-frozen in Luria Bertani (LB) (Thermo Fisher Scientific, United States) broth medium supplemented with 30% glycerol at −80°C for later use. *Pseudomonas aeruginosa* ATCC 27853 and *E. coli* ATCC 25922 were purchased as quality controls from the National Center for Clinical Laboratories (NCCL).

### Antibiotics and solvent

Naringenin was purchased from MedChemExpress (MCE) Co., Ltd. (NJ, United States). The antibiotics used in this study, including amikacin, ceftazidime, cefepime, imipenem, ciprofloxacin, aminotransim, levofloxacin, gentamicin, tobramycin, and colistin, were purchased from Wenzhou Kangtai Biotechnology Co., Ltd., Zhejiang China.

### Antimicrobial susceptibility testing

The minimum inhibitory concentration (MIC) of NG and commonly used antibiotics on 21 clinical GNB isolates was determined by the micro-broth dilution method with a simple modification (Andrews, 2001; Ogunniyi et al., 2017). A 96-well flat-bottomed microtitration plate with diluted antibiotics was prepared, and 100 μl of bacteria suspension were added to each well of the plate and incubated at 37°C for 16–20 h. Breakpoints for antibiotics were interpreted according to the CLSI guidelines. Each experiment was performed independently three times.

### Checkerboard assays

In total, 21 Col-R GNB isolates were selected for the checkerboard assays, in which effects of twofold serial dilutions of drug positioned in a 12 × 8 matrix were tested (MacNair et al., 2018; Zhang et al., 2021c). Each drug was diluted in cation-adjusted Mueller-Hinton broth (CAMHB) to a range of MIC concentrations for each experimental strain. Single bacterial colonies were cultured overnight and then diluted to the 0.5 McFarland standard in sterile saline. Next, samples were diluted in CAMHB in a 1:100 ratio. The final bacterial concentration in each well was approximately 7.5 × 10^5^ CFU/ml. The results were observed at 16–20 h at 37°C. The absorbance of the bacterial culture at 600 nm was measured with a microplate reader. All experiments were performed in triplicate with the average used for FIC calculations. The synergistic effect of NG in combination with colistin was assessed by using the fractional inhibition concentration index (FICI). The formula for calculating FICI is as follows: FICI = (MIC of drug A in combination/MIC of drug A alone) + (MIC of drug B in combination/MIC of drug B alone), where FICI ≤ 0.5 indicates synergism; FICI > 0.5 and ≤ 4 indicate irrelevant; and FICI > 4 was considered antagonistic (Fadwa et al., 2021).

### Time-kill assay

The synergistic effect of NG and colistin was further assessed using a time-killing assay based on the published articles with simple modifications (Berard et al., 2016). Six Col-R isolates (including two *K. pneumoniae*, two *E. coli*, and two *A. baumannii*) were selected to investigate their synergistic effects. Tubes containing LB alone served as the negative control. Briefly, bacteria were exposed to NG and colistin alone or NG + colistin at 1 × 10^6^ CFU/ml. The culture was shaken at 200 rpm at 37°C, and the number of bacteria was counted on LB agar plates at 0, 2, 4, 6, 12, and 24 h. The synergistic effect was defined as a reduction of 2 log_10_ CFU/ml (Fadwa et al., 2021). All studies were conducted in duplicate.

### Propidium iodide staining

The propidium iodide (PI) staining method was used to determine cell membrane permeability with a few modifications (Liu et al., 2017). Exponential phase cells of *K. pneumoniae* FK1913 were treated with test drugs alone (colistin 2 μg/ml; NG 64 and 128 μg/ml) or in combination for 2 h and placed in PI (50 μg/ml) for 10 min at room temperature. A fluorescent microscope (Nikon, Japan) was used to investigate the fluorescence at 561 nm, and photographs were recorded.

### Biofilm formation inhibition test

The effect of NG combined with colistin on biofilm formation was investigated by biofilm formation inhibition assays, with some minor modifications (Chen et al., 2021). Col-R *K. pneumoniae* (*n* = 3), *E. coli* (*n* = 3), and *A. baumannii* (*n* = 3) were selected for the experiment. First, to stimulate biofilm formation in bacteria, the starter cultures were diluted in 0.25% glucose-containing LB medium. Next, the strain was adjusted to 0.5 McFarland turbidity with sterile saline and then diluted to 1:100 in LB broth and transferred into 96-well plates. NG and colistin were added to the 96-well plates at final concentrations of 16–128 and 0.25–1 μg/ml, respectively. After incubation at 37°C for 24 h, the 96-well plates were washed three times with sterile phosphate-buffered saline (PBS) (Sigma-Aldrich, Milan, Italy) to remove planktonic bacteria. Next, the plates were dried at room temperature and incubated for 15 min at 37°C with 1% crystalline violet (Beijing Sun Biotechnology Co., Ltd., Beijing, China). The CVs were removed after staining, and the wells were washed three times with 1 × PBS. After that, 200 μl of ethanol-acetone were used to solubilize the bound CV (96:5 v/v), and the absorbance values taken at 595 nm were measured on an enzyme marker (Multiskan FC). All tests were performed in triplicate.

### Scanning electron microscopy

The biofilm inhibitory effect of NG combined with colistin was further investigated by scanning electron microscopy (SEM) (Zhang et al., 2021b). Reference experiments were performed with simple modifications. Briefly, FK1913 was randomly selected as the experimental strain. Overnight cultures of FK1913 were adjusted to 0.5 McFarland by sterile saline. Round sterile coverslips were placed in six-well cell culture plates. Diluted cultures (100 μl) were aliquoted into each well of a six-well plate containing 1,900 μl NG (64 μg/ml) and colistin (2 μg/ml) alone or in a combination of the two and incubated for 18–24 h at 37°C in a constant temperature incubator. The culture solution was aspirated from the wells, and the planktonic bacteria were washed away with PBS. Then, coverslips were fixed containing 2.5% glutaraldehyde for 4 h at 4°C. The samples were dried at 37°C for 15 min and then observed by SEM (S-3000N, Japan).

### *In vivo* evaluation of synergy in *Galleria mellonella* infection model

We investigated the *in vivo* efficacy of combination of NG and colistin by calculating the survival rate of the *Galleria mellonella* (*G. mellonella*) (Zhang et al., 2021c). As previously mentioned with simple modifications, *K. pneumoniae* (FK1913), *E. coli* (DC90), and *A. baumannii* (BM2349) were randomly selected as experimental strains. A single colony was selected and adjusted to 0.5 McFarland. FK1913 and DC90 were diluted to 1 × 10^6^ CFU/ml, and BM2349 was diluted to 1 × 10^7^ CFU/ml.

Insects weighing between 250 and 350 mg were chosen. Insects were then divided into four groups, namely, control group, NG monotherapy group, colistin monotherapy group, and combined drug treatment group. First, 10 μl of the bacterial solution was aspirated and injected into the left lower foot. The control group was only injected with a bacterial solution; the colistin single-drug group was injected with bacterial suspension and colistin (2, 4 μg/ml × 7); the NG group was injected with bacterial suspension and NG (16, 64 μg/ml × 7); the combination group was injected with the bacterial suspension and NG + colistin (2 + 64 μg/ml × 7, 4 + 64 μg/ml × 7, and 2 + 16 μg/ml × 7, respectively). Each group was injected with the bacterial suspension 2 h before antibiotic administration. The larvae were incubated at 37°C, and the survival rate was recorded for 7 days. When larvae do not respond to stimuli, they are considered to be dead. The survival rate of *G. mellonella* was analyzed by Kaplan-Meier analysis and log-rank test. All experiments were repeated three times.

### *In vivo* evaluation of synergy in the mouse infection model

To further evaluate the *in vivo* efficacy of NG and colistin, the neutropenic mouse thigh infection model was established with slight modifications (Lin et al., 2021). Female ICR mice, 5–6 weeks old (Charles River, Hangzhou, China), were used. Mice were cared for in accordance with the China’s National Standards for Laboratory Animals (GB 14925–2010). All animal studies were approved by the Zhejiang Association for Science and Technology (ID: SYXK [Zhejiang] 2018-0017) and carried out according to the guideline of the Wenzhou Laboratory Animal Welfare and Ethics.

FK1913 was randomly selected as the experimental strain. Neutropenic (neutrophils ≤100/mm^3^) thigh infections were induced by intraperitoneal injection of cyclophosphamide (Yuanye Biotechnology Co., Ltd., Shanghai, China) at 4 days (150 mg/kg) and 1 day (100 mg/kg). Each mouse was injected with 100 μl of bacterial suspension (1.5 × 10^7^ CFU/ml) in the posterior thigh muscle index. After 2 h of bacterial inoculation, colistin 7.5 mg/kg was injected intraperitoneally every 24 h, alone or in combination with NG (50 mg/kg every 24 h) (Jia et al., 2022). Mice were euthanized after 24 h of treatment. Bacterial load was quantified by CFU counts of post-thigh homogenates. Groups of three mice (six thigh infections) were included in each dosing regimen.

### *In vitro* cytotoxicity assays

To investigate the safety of NG, we performed a cytotoxicity assay using RAW264.7 with a few modifications (Chen et al., 2021). Briefly, RAW264.7 cells were cultured in Dulbecco’s Modified Eagle Medium (DMEM) supplemented with 10% heat-inactivated fetal bovine serum (FBS) and incubated in a 5% CO_2_ incubator at 37°C until fusion. Trypsin treatment was used to harvest confluent cells. Three groups were set up in the experiment, namely, the control group, the NG group, and the combined group. First, a 100 μl cell suspension containing 1 × 10^5^ cells was inoculated into a sterile 96-well plate. Then, 10 μl of different concentrations of NG (16, 32, 64, 128, 256, and 512 μg/ml) were added to the single-drug group. The combination group added 5 μl of NG and 5 μl of colistin (1 + 16 μg/ml, 1 + 32 μg/ml, 1 + 128 μg/ml, 2 + 64 μg/ml, 4 + 32 μg/ml, 4 + 64 μg/ml). The group without reagents was used as a control group. After that, the plate was incubated for 12 h. Next, 10 μl CCK-8 was added to each well and incubated for 1 h at room temperature in dark. Finally, the absorbance was recorded at 450 nm using a microplate reader.

### Statistical analysis

The results were expressed as mean ± standard deviation. The significance was determined using Student’s *t*-test and log-rank test. For all analyses, *P < 0.05, ^**^P < 0.01, and ^***^P < 0.001. Statistical analysis was performed using Prism 8.

## Results

### Antimicrobial susceptibility assay

The MICs of different isolates against common clinical antibiotics and NG are listed in Table 1. Most strains showed an MDR phenotype of 95.2% (20/21). The MICs of colistin against all strains were 4–64 μg/ml. The MICs of all strains for NG were > 512 μg/ml, which suggested that NG has no antibacterial activity against Col-R GNB.

**TABLE 1:** Minimum inhibitory concentration (MIC) values of commonly used clinical antibiotics and NG against 21 colistin-resistant GNB.

Species	Strains	Antibiotics	ATM	CAZ	FEP	IPM	CIP	LVX	GEN	TOB	COL	NG
		Breakpoints (S-R)	8–32	8–32	8–32	2–8	0.5–2	1–4	4–16	4–16	2–4	
*K. pneumoniae*	**FK1913**	MIC (μg/ml)	**≥256**	**≥256**	**≥256**	**32**	**≥256**	**128**	**≥256**	**≥256**	**>64**	**>512**
	**FK6663**		**≥256**	**≥256**	**≥256**	**32**	**≥256**	**≥256**	**≥256**	**≥256**	**8**	**>512**
	**FK169**		**≥256**	**≥256**	**≥256**	**32**	**128**	**32**	**128**	**64**	**>64**	**>512**
	**FK6556**		**64**	**64**	**64**	**16**	**4**	**8**	**16**	**16**	**16**	**>512**
	**FK1342**		**128**	**≥256**	**≥256**	0.25	**1**	0.5	1	4	**≥64**	**>512**
	FK1986		0.0125	0.25	0.0125	0.25	0.0125	0.025	2	1	**8**	**>512**
	**FK3994**		**≥256**	**128**	**≥256**	**32**	**≥256**	**64**	**≥256**	**≥256**	**64**	**>512**
*E. coli*	**DC3846**	MIC (μg/mL)	**128**	**64**	**≥256**	0.5	**≥256**	**128**	**≥256**	**64**	**8**	**>512**
	**DC7333**		**≥256**	**≥256**	**≥256**	**16**	**≥256**	**128**	**128**	**≥256**	**8**	**>512**
	**DC3539**		**64**	**128**	**≥256**	0.5	**64**	**16**	**128**	**128**	**8**	**>512**
	**DC3806**		**64**	**64**	**16**	1	**4**	**8**	**16**	**16**	**8**	**>512**
	**DC5286**		**≥256**	**128**	**≥256**	0.25	**128**	**64**	4	4	**8**	**>512**
	**DC90**		**≥256**	**32**	**64**	**≥256**	**64**	**32**	**≥256**	**128**	**4**	**>512**
	**DC3737**		**64**	**64**	**64**	**16**	**4**	**8**	**16**	**16**	**8**	**>512**
*A. baumannii*	**BM2349**	MIC (μg/ml)	**64**	**64**	**64**	**64**	**32**	**8**	**64**	**64**	**4**	**>512**
	**BM2431**		**64**	**64**	**64**	**16**	**4**	**8**	1	1	**32**	**>512**
	**BM2412**		**16**	**64**	**64**	**16**	**4**	**8**	4	1	**16**	**>512**
	**BM2370**		8	**32**	**128**	**8**	**128**	**8**	4	1	**8**	**>512**
	**BM2622**		**64**	**64**	**64**	**8**	**4**	**8**	**16**	**16**	**4**	**>512**
	**BM1539**		**16**	**8**	**64**	**16**	**4**	**2**	1	1	**8**	**>512**
	**BM1595**		2	**32**	8	**4**	**64**	**8**	**128**	**128**	**8**	**>512**

Bold strain number indicates multidrug-resistant (MDR) strain; Bold MIC values mean resistance.

GNB, gram-negative bacteria; MIC, minimum inhibitory concentration; ATM, aztreonam; CAZ, ceftazidime; FEP, cefepime; IMP, imipenem; CIP, ciprofloxacin; LVX, levofloxacin; GEN, gentamicin; TOB, tobramycin; COL, colistin; NG, naringenin; S, susceptible; R, resistance.

### Checkerboard assays

The MICs of colistin and NG were tested against a group of 21 strains of three different GNB. As shown in Table 2 and Supplementary Figure 1, the MIC of the Col-R strain was between 4 and 64 μg/ml. The MIC value of NG was >512 μg/ml. Mechanisms of colistin resistance were identified in these studied strains by the experimental team (Chen et al., 2020; Zhang et al., 2021c). The resistance mechanism of *E. coli* to colistin was attributed to *mcr-1* mobile colistin resistance gene. *K. pneumoniae* mediated colistin resistance through substitution of MgrB, PmrB, and PhoQ. In addition, *mcr-1* was also detected in four strains of *K. pneumoniae*. *A. baumannii* mediated colistin resistance by substituting LpxA, LpxC, LpxD, PmrB, and upregulation of the AdeABC/AdeIJK efflux pump. Checkerboard analysis showed that the combination of colistin and NG showed a significant synergistic effect on Col-R strains with different colistin resistance mechanisms (FICI ≤ 0.5), and the synergistic effect was independent of the resistance mechanism. In addition, MIC values of NG and colistin were significantly reduced when colistin was used in combination with NG. More importantly, the presence of NG resulted in the change of Col-R phenotype to colistin-sensitive phenotype in all strains.

**TABLE 2:** Combination activities of NG with colistin.

Species	Strains	Mechanism of colistin resistance	Monotherapy (μg/ml)		Combination (μg/ml)		FICI	Interpretation
			COL	NG	COL	NG		
*K. pneumoniae*	FK1913	MgrB (K2E, F28C), PhoQ (D150G)	>64	>512	2	64	<0.155	Synergistic
	FK6663	PmrB (R256G), *mcr-1*	8	>512	2	64	<0.375	Synergistic
	FK169	MgrB (K2E, F28C)	>64	>512	1	128	<0.265	Synergistic
	FK6556	PmrB (R256G), *mcr-1*	16	>512	2	128	<0.375	Synergistic
	FK1342	MgrB (K2E, F28C), PhoQ (D150G), *mcr-1*	>64	>512	2	128	<0.281	Synergistic
	FK1986	MgrB (K2E, F28C), PhoQ (D150G)	8	>512	2	64	<0.375	Synergistic
	FK3994	PmrB (R256G), *mcr-1*	64	>512	2	64	<0.155	Synergistic
*E. coli*	DC3846	*mcr-1*	8	>512	1	128	<0.375	Synergistic
	DC7333	*mcr-1*	8	>512	1	128	<0.375	Synergistic
	DC3539	*mcr-1*	8	>512	1	128	<0.375	Synergistic
	DC3806	*mcr-1*	8	>512	2	64	<0.375	Synergistic
	DC5286	*mcr-1*	8	>512	2	64	<0.375	Synergistic
	DC90	*mcr-1*	4	>512	1	64	<0.375	Synergistic
	DC3737	*mcr-1*	8	>512	1	128	<0.375	Synergistic
*A. baumannii*	BM2349	PmrB (N163I), AdeABC/AdeIJK	4	>512	1	16	<0.28	Synergistic
	BM2431	PmrB (I10T), AdeABC/AdeIJK	32	>512	0.5	32	<0.077	Synergistic
	BM2412	LpxC (P46Q), AdeABC/AdeIJK	16	>512	1	32	<0.123	Synergistic
	BM2370	PmrB (W11R), LpxD (N148K), AdeABC/AdeIJK	8	>512	0.5	16	<0.09	Synergistic
	BM2622	LpxC (S186R), LpxD (T289I), PmrB (A138T), AdeABC/AdeIJK	4	>512	0.25	32	<0.123	Synergistic
	BM1595	PmrB (N163I), AdeABC/AdeIJK	8	>512	1	16	<0.156	Synergistic
	BM1539	LpxA (A182V), LpxC (P46Q), AdeABC/AdeIJK	8	>512	1	32	<0.187	Synergistic

FICI, fractional inhibitory concentration index; NG, Naringenin; COL, colistin.

### Time−kill assay

**FIGURE 1:**
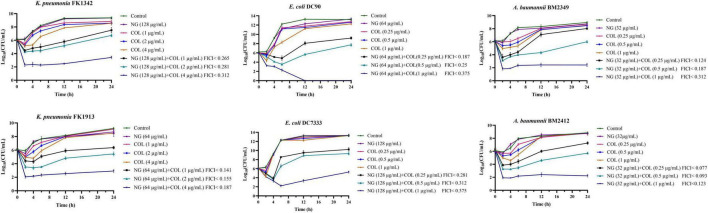
Colistin and naringenin time-killing curves against colistin-resistant *Klebsiella pneumonia*, colistin-resistant *Escherichia coli*, and colistin-resistant *Acinetobacter baumannii* when used alone or in combination. Naringenin (NG) and colistin (COL).

As shown in Figure 1, the synergistic effect of NG and colistin on Col-R GNB was further analyzed using a time-kill curve. Drug concentrations were selected from results determined by the checkerboard method with FICI < 0.5. The experimental results showed that the monotherapy group had fewer time-dependent bactericidal activities than the control group. In addition, NG in combination with higher concentrations of colistin resulted in faster killing, and the strains treated with the combination exhibited a significant reduction in viable cells [more than >3log_10_ (CFU/ml)] compared with the control group. In summary, NG and colistin had very strong bactericidal activity when used together.

### Mechanisms for drug synergy

**FIGURE 2:**
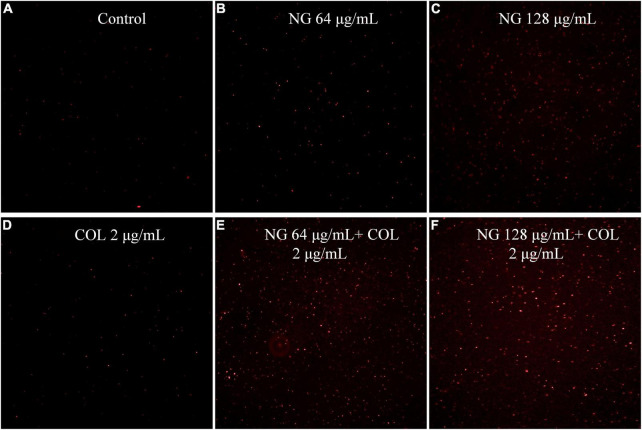
Fluorescence microscopy imaging of exponential-phase *Klebsiella pneumonia* FK1913, which were treated with naringenin and colistin alone or in combination, and incubated with 50 μg/ml PI for 10 min before imaging. **(A)** LB broth control; **(B,C)** cells treated with naringenin at 64, 128 μg/ml; **(D)** cells treated with colistin with 2 μg/ml; **(E,F)** cells exposed to a combination of naringenin and colistin. Naringenin (NG) and colistin (COL).

We evaluated the cell membrane permeability of *K. pneumoniae* FK1913 using PI. As revealed by fluorescence microscopic analysis (Figure 2), after pre-incubating cells with colistin at 2 μg/ml, a minimal effect on cell membrane permeability was observed. However, pre-incubation of the cells with NG resulted in a concentration-dependent enhancement in fluorescence intensity due to PI uptake and DNA binding, which indicated that the integrity of the cell membrane gradually decreased.

### The antibiofilm activity of colistin/naringenin combinations

**FIGURE 3:**
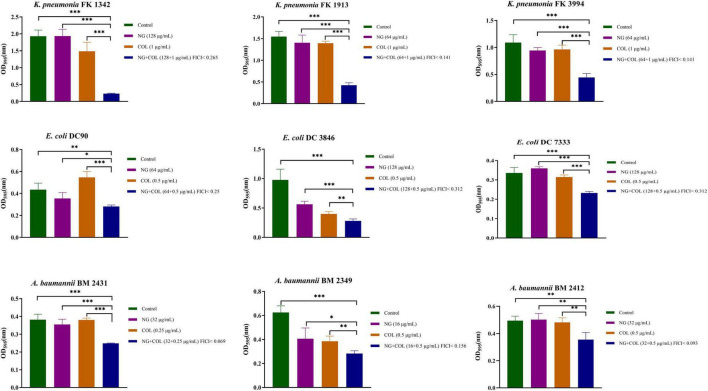
Biofilm inhibitory effects of colistin combined with naringenin on colistin-resistant *Klebsiella pneumonia*, colistin-resistant *Escherichia coli*, and colistin-resistant *Acinetobacter baumannii*. Drug concentration was chosen from the checkerboard method with FICI < 0.5. *P < 0.05, **P < 0.01, and ***P < 0.001 analyzed by Student’s *t*-test. Naringenin (NG) and colistin (COL).

We investigated the ability of NG and colistin to inhibit biofilms when used alone or in combination. As shown in Figure 3, NG + colistin inhibited the biofilm of Col-R GNB compared with the blank control and NG or colistin alone (*P* < 0.05). The concentrations selected were derived from the synergistic concentrations of each strain in the checkerboard method.

### Scanning electron microscopy

**FIGURE 4:**
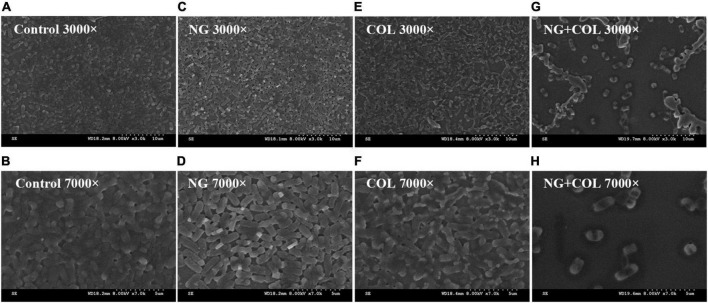
Scanning electron microscopy image. *Klebsiella pneumonia* FK1913 treated with 64 μg/ml naringenin **(C,D)**, 2 μg/ml colistin **(E,F)**, or combined treatment **(G,H)** for 2 h. **(A,B)** The control condition. Naringenin (NG) and colistin (COL).

To further demonstrate the inhibitory effect of NG/colistin combination on biofilms, SEM experiments were performed. We selected a strain of Col-R *K. pneumoniae* FK1913 for observation. As shown in Figure 4, the results showed that untreated *K. pneumoniae*, treated with NG (64 μg/ml) and colistin (2 μg/ml) alone, exhibited a dense number of biofilms and complete morphology. However, the combination of NG and colistin greatly reduced the number of biofilms and formed a single colony. It is further proved that the combination of NG and colistin can inhibit the formation of biofilm.

### *In vivo* study

**FIGURE 5:**
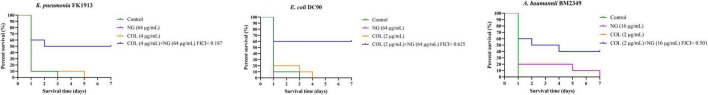
Survival rate of *Galleria mellonella* after 7 days of monotherapy or combination therapy against colistin-resistant *Klebsiella pneumoniae* FK1913, *Escherichia coli* DC90, and *Acinetobacter baumannii* BM2349. Naringenin (NG) and colistin (COL).

To demonstrate the combined effect of NG and colistin *in vivo*, we conducted the *G. mellonella* test. We selected one strain of Col-R *K. pneumoniae*, *E. coli*, and *A. baumannii* for the experiment. As shown in Figure 5, almost all strains of *G. mellonella* injected with only the bacterial suspension died after 72 h. The survival rate of *G. mellonella* was significantly lower with monotherapy than with combination therapy (*P* < 0.05).

**FIGURE 6:**
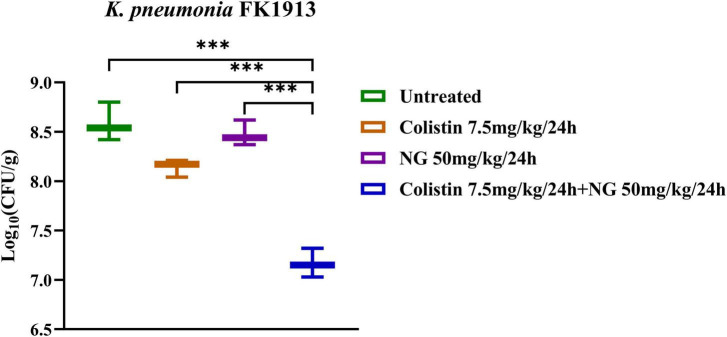
Changes in Log_10_ of thigh muscle (Δlog_10_ CFU/thigh) 24 h after monotherapy (naringenin 50 mg/kg, colistin 7.5 mg/kg) or combination therapy (50 mg/kg NG + 7.5 mg/kg colistin) for colistin-resistant *Klebsiella pneumoniae* FK1913. Naringenin (NG) and colistin (COL). ***P < 0.001.

Meanwhile, the *in vivo* efficacy of the NG/colistin combination was further evaluated by a mouse thigh infection model. As shown in Figure 6, **50** mg/kg NG and 7.5 mg/kg colistin had a slight inhibition of *K. pneumoniae* FK1913. In addition, NG combined with colistin showed a higher efficacy for 24 h compared with NG and colistin alone (*P* < 0.05). *In vivo* experiments showed that the combination of the two drugs had a significant synergistic antibacterial effect.

### *In vitro* evaluation of cytotoxicity

**FIGURE 7:**
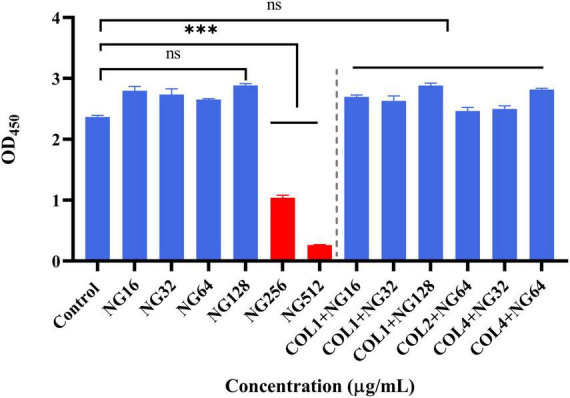
Cytotoxic effect of naringenin alone and in combination with different concentrations against the RAW 264.7 murine macrophage cell line (absorbance values at 450 nm). Data were analyzed by Student’s *t*-test; ns, not statistically significant; ***P < 0.001. Naringenin (NG) and colistin (COL).

To verify the safety of NG, we investigated the potential toxicity of NG alone and in combination on RAW264.7 cells. As depicted in Figure 7, the results indicated that NG concentration was 128 μg/ml without a toxic effect on RAW264.7 cells compared with the CCK-8 treated group. In addition, all concentrations of NG and colistin combined in this experiment showed no cytotoxic effects. This result demonstrates that the combined regimen can be used within a safe range in humans.

## Discussion

In recent years, the massive and inappropriate use of broad-spectrum antibiotics has led to the emergence of MDR GNB (Rice, 2009). These drug-resistant pathogens pose a serious threat to the health and safety of hospitalized patients, as they are usually associated with a higher mortality rate (Brummett and Fox, 1989). Colistin has been used as a “last resort” to treat GNB infections. However, with its widespread use, the number of Col-R strains has significantly increased (Cannatelli et al., 2013; Olaitan et al., 2014). Therefore, developing and discovering new therapeutic means to treat these Col-R GNB strains remain crucial (Bush, 2010). To the best of our knowledge, this is the first study that reported how NG combined with colistin exhibited good synergistic effects *in vivo* and *in vitro*, thus providing a new therapeutic option for clinical Col-R GNB infections.

Global data indicate that colistin resistance is rising, especially with the discovery of *mcr-1* plasmid found worldwide (Wang et al., 2018b). Previous studies have shown that colistin has significant neurotoxicity and nephrotoxicity and has been abandoned for use (Fiaccadori et al., 2016). Therefore, it is extremely important for us to restore the activation of colistin and reduce the dosage of colistin. In this study, we used the checkerboard method and found that most strains have an MDR phenotype of 95.2% (20/21), and that colistin shows different drug resistance mechanisms. The presence of NG significantly reduces the MIC value of colistin, which manifests as FICI < 0.5, and restores the colistin sensitivity of GNB with different colistin resistance mechanisms. The time-killing assay confirmed further that the combined use of NG and colistin reduces the number of bacteria compared with a single drug, confirming synergistic effect of NG and colistin on Col-R GNB.

In addition, the inhibition test of biofilm formation showed that the combination of NG and colistin could inhibit the biofilm formation of Col-R GNB. To further prove the combination of the two effects on biofilm formation, we randomly selected a Col-R GNB strain (FK1913) for SEM. The results showed no change in biofilm morphology when NG or colistin was used alone. However, the combination of colistin and NG significantly disrupted the formation of *K. pneumoniae* biofilm, and the number of bacteria was significantly reduced. In this study, NG alone did not significantly inhibit the biofilm of GNB. We speculate that this may be due to the concentration of NG used in this study, which was not the concentration required to inhibit GNB.

To investigate the effects of NG and colistin *in vivo*, we used the *G. mellonella* test and mouse thigh infection model. The results showed that NG combined with colistin could significantly improve the survival rate of *G. mellonella*. Notably, NG combined with colistin significantly reduced the number of bacteria in mice. NG has been reported to increase membrane permeability and change the morphology of *Staphylococcus aureus* (Wang et al., 2017). On this basis, we further explored the synergistic mechanism of NG and colistin by PI staining. The results showed that NG could overcome colistin resistance by increasing membrane permeability, which allows more colistin to cross cell membranes and act as an antibacterial agent.

More importantly, the safety of NG was further confirmed by cytotoxicity assay. The results showed that 128 μg/ml NG alone had no cytotoxicity on RAW264.7 cells, and none of the combination concentrations used in this study showed any cytotoxicity. *G. mellonella* test, the mouse thigh infection model, and cytotoxicity test proved that NG combined with colistin is safe and effective. The safety and pharmacokinetics of NG have also been reported, and clinical trials have demonstrated the safety of 900 mg NG (Rebello et al., 2020). Especially now that coronavirus disease 2019 (COVID-19) is still at a critical juncture, it has been demonstrated that NG may have a therapeutic effect on COVID-19 by inhibiting COVID-19 major protease, 3CLpro, and reducing ACE2 receptor activity (Tutunchi et al., 2020). This suggests that a combination of NG and colistin might be a feasible option for patients with both COVID-19 infection and Col-R GNB. The potential medical value of NG was verified. NG has been shown to reduce methotrexate-induced pancytopeni, which is both nephrogenic and hepatotoxic (Dhanisha et al., 2022).

In summary, our study indicates that NG combined with colistin has a significant synergistic effect on Col-R GNB with different colistin resistance mechanisms. Furthermore, this study highlights the potential for reusing colistin and restoring the activity that maintains the last line of colistin. More importantly, NG combined with colistin can reduce the dose of colistin, thereby reducing its side effects of colistin (neurotoxicity and nephrotoxicity), and still maintaining the maximum therapeutic effect. Therefore, this study has great significance for the limited use of colistin in treating Col-R GNB bacterial infection, providing a prospective option for future treatment.

## Data availability statement

The raw data supporting the conclusions of this article will be made available by the authors, without undue reservation.

## Ethics statement

The animal study was reviewed and approved by the ethics committee of the Wenzhou Medical University (SYXK 2020-0014).

## Author contributions

MX wrote the first draft of the manuscript. MX, ZY, YZ, and SS prepared the material and collected the data. YS, LF, and XZ analyzed the data. CZ, JC, and TZ supervised the research. All authors contributed to the revisions of the manuscript and approved the final manuscript.

## Supplementary material

Supplementary Figure 1 Checkerboard analysis of the synergistic antibacterial activity of the combination of colistin and naringenin against colistin-resistant *Klebsiella pneumonia*, colistin-resistant *Escherichia coli*, and colistin-resistant *Acinetobacter baumannii*. The absorbance of the bacterial cultures at 600 nm is shown at 16–20 h incubation. The dark blue area represents higher cell density. Data represent the mean OD_600_ of three biological replicates. Naringenin (NG) and colistin (COL).
