# Supplementary figures and images for: Naringenin restores colistin activation against colistin-resistant gram-negative bacteria in vitro and in vivo
Source: Front Microbiol. 2022 Aug 3;13:916587. doi: 10.3389/fmicb.2022.916587 (PMC9382302; doi:10.3389/fmicb.2022.916587)

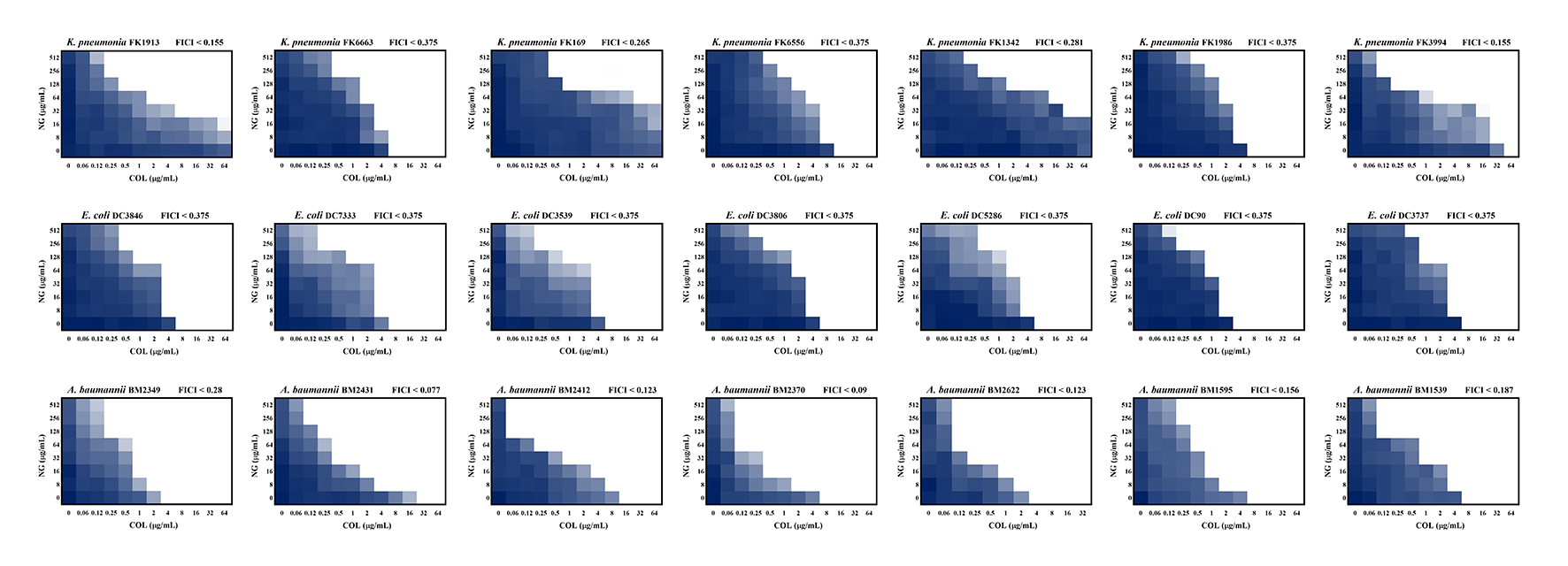

Supplement: Supplementary Figure 1 — Checkerboard analysis of the synergistic antibacterial activity of the combination of colistin and naringenin against colistin-resistant Klebsiella pneumonia, colistin-resistant Escherichia coli, and colistin-resistant Acinetobacter baumannii. The absorbance of the bacterial cultures at 600 nm is shown at 16–20 h incubation. The dark blue area represents higher cell density. Data represent the mean OD600 of three biological replicates. Naringenin (NG) and colistin (COL). [file Image_1.TIF]
